# Integrating Phosphatidylethanol to Enhance Alcohol Quantification in Steatotic Liver Disease and Clinical Trials

**DOI:** 10.1111/liv.70489

**Published:** 2025-12-22

**Authors:** Elias D. Rady, Thomas G. Cotter

**Affiliations:** ^1^ Division of Digestive and Liver Diseases UT Southwestern Medical Center Dallas Texas USA; ^2^ Evidentis Clinical Research, Fort Worth Texas USA

**Keywords:** ALD: alcohol‐associated liver disease, AUDIT‐C: alcohol use disorders identification test–consumption, MASLD: metabolic dysfunction‐associated Steatotic liver disease, MetALD: metabolic dysfunction and alcohol‐associated liver disease, PEth: phosphatidylethanol, SLD: steatotic liver disease, TLFB: Timeline Followback

## Abstract

Accurate quantification of alcohol intake is essential for the diagnosis, subclassification, and management of steatotic liver disease (SLD). In everyday clinical practice, alcohol histories rely primarily on physician‐assisted self‐report, with validated questionnaires used variably. Both provider‐level inconsistencies in documentation and patient‐level factors (e.g., recall and social‐desirability biases) can contribute to misclassification of SLD subtypes. Phosphatidylethanol (PEth), a direct alcohol biomarker detectable for ~2–4 weeks, has emerged as a promising objective tool that can complement self‐report. Across diverse SLD populations, PEth consistently uncovers underreported alcohol use, sharpens subclassification, informs prognosis, and enables longitudinal monitoring to guide care. In liver transplantation, PEth supports documentation of abstinence and detection of relapse to enable timely intervention. This mini‐review synthesises current evidence on PEth in SLD, emphasises its value in both clinical and research settings, and outlines priorities for integrating PEth into structured care pathways and clinical trial design to improve outcomes and harmonise endpoints.

## Key Points

- Accurate alcohol quantification is essential for correct subclassification and management of steatotic liver disease (SLD).
- Self‐report is prone to provider‐level documentation inconsistencies and patient‐level biases, contributing to misclassification of MASLD, MetALD, and ALD.
- Phosphatidylethanol (PEth) is a direct, highly specific biomarker that detects alcohol use over the prior 2–4 weeks.
- PEth improves diagnostic accuracy, refines prognostication, and enables objective longitudinal monitoring in clinical and research settings.
- Implementing serial PEth lead‐in screening with ongoing monitoring and control of disease modifiers in trial design ensures accurate classification, consistent intake, and interpretability of trial endpoints.

**Abbreviations**
AEs: adverse events
ALD: alcohol‐associated liver disease
ALT: alanine aminotransferase
AST: aspartate aminotransferase
AUD: alcohol use disorder
AUDIT‐C: alcohol use disorders identification test–consumption
CDT: carbohydrate‐deficient transferrin
CMRFs: cardiometabolic risk factors
EtG: ethyl glucuronide
EtS: ethyl sulfate
FAEEs: fatty acid ethyl esters
FGF21: fibroblast growth factor 21
GGT: gamma‐glutamyl transferase
GLP‐1 RAs: glucagon‐like peptide‐1 receptor agonists
LT: liver transplantation
MASLD: metabolic dysfunction‐associated steatotic liver disease
MCV: mean corpuscular volume
MELD: model for end‐stage liver disease
MetALD: metabolic dysfunction and alcohol‐associated liver disease
PEth: phosphatidylethanol
SAEs: serious adverse events
SLD: steatotic liver disease
TLFB: Timeline Followback

## 1 Introduction

Alcohol remains a leading cause of liver‐related morbidity and mortality worldwide, contributing substantially to hospital encounters and the overall burden of liver disease [1]. Within the recently proposed steatotic liver disease (SLD) nomenclature, alcohol consumption is a central determinant of disease subtype [2]. The introduction of the metabolic dysfunction and alcohol‐associated liver disease (MetALD) category—positioned between metabolic dysfunction‐associated steatotic liver disease (MASLD) and alcohol‐associated liver disease (ALD)—underscores the synergistic and overlapping effects of metabolic dysfunction and alcohol exposure on disease progression. To date, alcohol documentation in routine practice relies primarily on physician‐assisted self‐report. Both provider‐level documentation inconsistencies and patient‐level factors (e.g., recall and social desirability biases) can contribute to misclassification of alcohol intake and, in turn, SLD subtypes [3, 4, 5]. This hasdirect implications for patient care, prognostic modelling, and trial validity, where accurate phenotyping is essential for risk stratification, therapeutic decision‐making, and establishing reproducible inclusion criteria.

To address these limitations, there is increasing interest in objective alcohol biomarkers to complement clinician histories and validated questionnaires. Among available biomarkers, phosphatidylethanol (PEth) has emerged as a leading candidate, offering high specificity and sensitivity, a detection window of up to 4 weeks, and compatibility with routine whole‐blood sampling [6]. Beyond its diagnostic and prognostic utility, PEth is being increasingly applied in SLD research, where reliable classification and longitudinal monitoring of alcohol use are critical for interpreting outcomes. This review examines the rationale for accurate alcohol quantification in SLD, summarises the biological and analytical basis of PEth, and evaluates current evidence supporting its integration into both clinical care and clinical trial design within the evolving SLD landscape.

## 2 The Need for Accurate Quantification of Alcohol Intake in SLD

In the current SLD framework, subtypes are defined by the presence of cardiometabolic risk factors (CMRFs) and alcohol intake thresholds. Patients with hepatic steatosis and at least one CMRF are classified as MASLD if alcohol intake is below 20 g/day for women or 30 g/day for men, as MetALD if between 20 and 50 g/day for women or 30–60 g/day for men, and as ALD if exceeding 50 g/day for women or 60 g/day for men [2]. In the absence of CMRFs, lower thresholds—above 20 g/day for women or 30 g/day for men—are used to define ALD. Classification often relies primarily on physician‐assisted self‐report, and International Classification of Diseases (ICD) codes, which are prone to documentation inconsistencies [3]. In fact, several studies highlight discrepancies between reported and actual intake. In a prospective Austrian cohort of presumed MASLD, hair ethyl glucuronide (EtG) testing revealed moderate‐to‐excessive intake (10–60 g/day) in 29% and severe harmful use (> 60 g/day) in 10% of participants [7]. Similarly, analysis of a Swedish registry of more than 15 000 individuals with MASLD found that 12% had a documented history of ALD or alcohol use disorder (AUD)—a subgroup with significantly higher rates of major adverse liver outcomes [8]. These findings suggest that a substantial proportion of patients categorised as MASLD would be more accurately classified as MetALD or ALD if alcohol intake were assessed objectively.

Validated questionnaires based on self‐report such as the Alcohol Use Disorders Identification Test—Consumption (AUDIT‐C) and Timeline Followback (TFLB) can complement physician histories [3]. The AUDIT‐C is short and commonly used as a screening tool in clinical practice but might not adequately assess sporadic drinking patterns. TLFB is used in alcohol research and provides daily estimates over a specific period but is time‐consuming in clinical practice. However, regardless of the method used, self‐report is inherently affected by patient‐level biases given the sensitive nature of alcohol use. Social desirability bias for example can drive conscious or unconscious underreporting [4], recall bias is particularly problematic for individuals with irregular or binge‐drinking patterns [5], while stigma may discourage disclosure due to concerns about judgement, discrimination, or negative repercussions for medical care, employment, or personal relationships [9].

These diagnostic and prognostic gaps underscore the urgent need for objective, standardised methods to complement self‐report histories and questionnaires. PEth has emerged as a leading biomarker with potential to reduce misclassification and improve both clinical management and research validity.

## 3 Biological and Analytical Basis of PEth

PEth is an atypical phospholipid formed when phospholipase D, in the presence of ethanol, catalyses a transphosphatidylation reaction substituting ethanol for water in phosphatidylcholine [6]. More than 40 homologues have been described, with PEth 16:0/18:1 the most abundant and clinically informative. Once incorporated into erythrocyte membranes, PEth accumulates with continued alcohol exposure and is eliminated slowly, with a biphasic half‐life of approximately 3–10 days. Depending on drinking pattern and intensity, it remains detectable for up to 4 weeks after cessation, providing a reliable measure of recent alcohol use over extended periods.

Quantification requires whole blood—not serum or plasma—because PEth resides within erythrocyte membranes [6]. Measurement is performed using liquid chromatography–tandem mass spectrometry (LC–MS/MS), which offers high sensitivity, specificity, and the ability to distinguish among molecular species. PEth 16:0/18:1 shows good short‐term stability in whole blood, but concentrations decline with storage at −20°C beyond ~28 days; therefore, −80°C is recommended for long‐term/biobank storage, with prompt analysis after thaw [10]. To harmonise interpretation, the 2022 Basel Consensus designated PEth 16:0/18:1 as the reference analyte and recommended standardised cutoffs: < 20 ng/mL (0.028 μmol/L) consistent with abstinence or low consumption, 20–200 ng/mL (0.028–0.28 μmol/L) reflecting moderate use, and > 200 ng/mL (> 0.28 μmol/L) suggesting chronic excessive intake [11]. These thresholds, though widely applied, are primarily consensus‐based and informed by correlations with self‐reported intake (often the TLFB). They have not been validated for subclassifying alcohol exposure within the SLD framework, where disease biology and outcomes may differ from populations in which these cutoffs were developed.

A notable advantage of PEth is that its concentrations are not significantly influenced by underlying liver disease, since formation occurs within erythrocyte membranes and elimination is independent of hepatic metabolism [12]. This contrasts with indirect biomarkers (e.g., GGT, CDT, MCV), which are often unreliable in advanced liver disease. Still, several factors can complicate interpretation: hemolysis may reduce concentrations, while recent transfusion can yield falsely elevated values. For these reasons, PEth should be regarded as a robust but complementary biomarker, best interpreted alongside clinician histories and other available tools for assessing alcohol intake (Table 1).

**TABLE 1:** Overview of self‐report and biomarker tools for assessing alcohol use in steatotic liver disease.

Method	Brief description	Detection window	Key strengths	Key limitations
*SELF‐report*				
AUDIT‐C	3‐item screening tool assessing alcohol consumption; score ≥ 3 (women) or ≥ 4 (men) suggests hazardous use	Past year	Quick; validated in liver disease; widely used.	Does not quantify exact intake; limited sensitivity for intermittent heavy use.
Timeline Followback (TLFB)	Calendar‐based recall anchored to personal/public events to reconstruct drinking history	1 month (commonly used in trials)	Provides detailed daily drinking patterns; improved recall with memory aids.	Time‐consuming; requires interviewer training.
*Direct biomarkers*				
PEth (Phosphatidylethanol)	Phospholipid formed only in presence of ethanol measured in whole blood	~2–4 weeks	High specificity/sensitivity; not affected by liver metabolism.	Influenced by hematologic factors; false positives after transfusion.
Ethyl glucuronide (EtG)—urine	Non‐oxidative ethanol metabolite detected in urine	Up to 3–5 days	High sensitivity for recent drinking.	Short window; incidental exposure can cause positives.
Ethyl glucuronide (EtG)—hair	EtG incorporated into hair shaft	Weeks–6 months	Long‐term intake patterns; useful for chronic monitoring.	Cannot detect recent use; affected by hair treatments.
Ethyl sulfate (EtS)—urine	Non‐oxidative ethanol metabolite often measured with EtG	Up to 3 days	Confirms EtG; reduces false positives.	Short window; low standalone utility.
Fatty acid ethyl esters (FAEEs)—hair/sebum	Formed by esterification of ethanol with fatty acids; deposited in hair/sebum.	Weeks–months	Complements hair EtG for chronic use.	Affected by cosmetics; less standardised.
*Indirect biomarkers*				
Carbohydrate‐deficient transferrin (CDT)	Serum glycoprotein isoform elevated after sustained heavy use (> 40–60 g/day)	2–3 weeks	Relatively high specificity for heavy use.	Low sensitivity in advanced liver disease; affected by genetic variants.
Gamma‐glutamyl transferase (GGT)	Liver enzyme induced by chronic alcohol intake	Weeks (reflects recent intake)	Widely available; inexpensive.	Poor specificity; elevated in many liver and non‐liver conditions.
Aspartate aminotransferase (AST)	Liver enzyme often higher than ALT in heavy use (AST:ALT > 2)	Variable	Commonly available; may suggest alcohol‐related injury.	Low specificity; elevated in many liver diseases.
Mean corpuscular volume (MCV)	Average red blood cell size; rises with sustained heavy drinking	Weeks–months	Simple, inexpensive; part of routine CBC.	Low specificity; affected by anaemia and other conditions.

Abbreviations: ALT, alanine aminotransferase; AST, aspartate aminotransferase; AUDIT‐C, alcohol use disorders identification test–consumption; CBC, complete blood count; CDT, carbohydrate‐deficient transferrin; EtG, ethyl glucuronide; EtS, ethyl sulfate; FAEEs, fatty acid ethyl esters; GGT, gamma‐glutamyl transferase; LC‐MS/MS, liquid chromatography–tandem mass spectrometry; MCV, mean corpuscular volume; PEth, phosphatidylethanol; TLFB, Timeline Followback.

## 4 Clinical and Research Applications of PEth in Steatotic Liver Disease

### 4.1 Identifying Under‐Reported Alcohol Use

Direct alcohol biomarkers such as PEth play an increasingly important role in refining SLD subclassification, given the consistent underestimation of alcohol consumption when relying on self‐report. In a U.S. retrospective study of 279 patients with chronic liver disease, concordance between self‐reported drinks and PEth levels was observed in only one‐third of patients, with 58% under‐reporting their alcohol intake [13]. Strikingly, among those reporting no or light drinking, 86% had PEth ≥ 20 ng/mL, indicating at least moderate use.

Similar discrepancies have been demonstrated in rigorously phenotyped cohorts. Among 120 patients with biopsy‐proven MASLD, 10% had PEth > 0.3 μmol/L (> 210 ng/mL), consistent with heavy use, suggesting that some patients classified as MASLD by history alone may actually meet ALD criteria [14]. In the San Diego Liver Study, 16% of 391 adults with overweight/obesity and SLD were identified as under‐reporters when comparing TLFB with PEth testing. Incorporation of PEth into diagnostic algorithms resulted in a fourfold increase in MetALD diagnoses and a threefold increase in ALD diagnoses compared with self‐report alone [15]. Similarly, in a cross‐sectional study of nearly 3000 individuals at risk for SLD, median PEth rose stepwise across MASLD, MetALD, and ALD, with 39.0% of those classified as MASLD by history having PEth ≥ 20 ng/mL, consistent with MetALD/ALD thresholds. Importantly, an AUDIT‐C–guided approach to PEth testing reduced testing by 43% while missing only ~3% of high‐PEth cases, supporting complementary use of AUDIT‐C and PEth rather than reliance on either alone [16].

Collectively, these studies indicate frequent underestimation of alcohol exposure. In routine hepatology, combining PEth with clinician history and validated questionnaires provides objective evidence of recent intake, improving SLD classification and prompting timely AUD interventions for newly referred chronic liver disease patients.

### 4.2 Proposed PEth Thresholds for SLD Subclassification

Several groups have evaluated PEth cutoffs for detecting elevated intake. In chronic liver disease, Stewart et al. validated 20 ng/mL as a specific cutoff for one‐month abstinence and 80 ng/mL as a sensitive threshold for > 4 drinks/day (> 56 g/day) [17]. In the San Diego Liver Study, a 25 ng/mL threshold best distinguished MASLD from MetALD, with ≥ 90% specificity at 35 ng/mL; applying 80 ng/mL corresponded to ≥ 56 g/day [18]. A Swedish population‐based cohort of > 6000 individuals with presumed MASLD used PEth 35–210 ng/mL to define MetALD, identifying ~20% under‐reporting [19]. In a Danish prospective cohort of 192 patients with SLD, self‐report correlated moderately with PEth (*r* = 0.617) and poorly with CDT (*r* = 0.316) [20]. Among 116 patients with alcohol‐related cirrhosis, PEth within a three‐week detection window demonstrated 77% sensitivity/90% specificity for any increased intake and 83%/81% for excessive intake [21].

Taken together, these data show the potential role of PEth in SLD trials in supplementing inclusion/exclusion criteria, improving stratification, and monitoring alcohol exposure during the study. To note, several cited cut‐offs come from single‐study contexts and diverge from consensus—the San Diego Liver Study had few PEth‐positive cases, and the Swedish population cohort lacked a biomarker reference standard—so these values are best treated as working thresholds, not final standards. Interpretation should also consider pattern and timing of drinking: intermittent or binge‐weighted use can yield lower steady PEth than near‐daily intake with similar total grams per week, whereas recent heavy days may raise levels because PEth forms in erythrocytes and clears over several days [22]. Therefore, there is a pressing need to build consensus on PEth cut‐offs through multicenter, outcome‐anchored studies to ensure generalizability and cross‐study comparability.

### 4.3 PEth for Monitoring Alcohol Intake

Alcohol use in SLD is highly dynamic, often fluctuating over weeks to months, which makes repeated assessment essential for accurate characterisation. In the Danish cohort of patients with SLD, serial evaluations over 12 months showed that 53% of those reporting intake below MASLD thresholds at baseline exceeded these thresholds at follow‐up, while 47% remained below [20]. These findings highlight the limitations of a single timepoint assessment and reinforce the need for longitudinal, objective monitoring rather than static classification.

PEth offers a sensitive and reproducible means of capturing these changes. Although the Danish study is the first to examine serial PEth assessments specifically in SLD, prior work in broader liver disease and AUD supports its role in longitudinal monitoring. In a prospective cohort of 64 patients with liver disease undergoing alcohol intervention, median PEth fell from 0.93 μmol/L at baseline to 0.09 μmol/L after 4 weeks of confirmed abstinence, correctly classifying 92% of abstinent and 88% of non‐abstinent participants compared with TLFB [23]. In a U.S. trial of heavy drinkers with AUD, median PEth levels decreased from 306 ng/mL to 140 ng/mL during an 11‐week contingency management program, with ≥ 50% weekly declines during abstinent periods and an AUC of 0.81–0.83 for predicting self‐reported drinking in the prior 1–2 weeks [24].

Taken together, these findings highlight the strength of PEth as a longitudinal monitoring tool, capable of capturing real‐world fluctuations in alcohol use that static measures cannot. Its value extends beyond confirming abstinence or categorising patients at a single timepoint—serial PEth testing enables clinicians and investigators to follow alcohol exposure trajectories, contextualise treatment responses, and operationalise alcohol as a quantitative co‐factor in both clinical practice and trial design.

### 4.4 Prognostic Value of PEth in SLD

Alcohol use is a key determinant of adverse outcomes across the SLD spectrum, even at modest levels. Among more than one million U.S. veterans with SLD, reducing high‐risk alcohol use (AUDIT‐C ≥ 3 in women, ≥ 4 in men) lowered cirrhosis risk by 39%, and sustained abstinence improved prognosis in 320 patients with alcohol‐related cirrhosis regardless of baseline portal hypertension [25].

PEth provides objective prognostic value by stratifying risk according to biochemical evidence of alcohol exposure. In the Swedish MASLD cohort, PEth 35–210 ng/mL was associated with a 3.7‐fold higher risk of major adverse liver outcomes, while PEth > 210 ng/mL conferred an 8.5‐fold higher risk, independent of metabolic and demographic factors [19]. In the Danish cohort, upward shifts in PEth category during follow‐up were independently linked to increased risk of hepatic decompensation or death (adjusted HR 2.38; 95% CI 1.09–5.19), whereas stable or declining levels correlated with improved outcomes [20].

These data support the role of PEth as a useful adjunct prognostic biomarker. By quantifying recent alcohol exposure on a continuum, PEth complements clinician history to refine risk prediction and to inform the timing and intensity of AUD‐directed care in practice, while enhancing endpoint attribution in trials.

### 4.5 Use of PEth in Liver Transplantation

Monitoring alcohol use before and after liver transplantation (LT) is critical, as return to drinking substantially affects graft function, patient survival, and long‐term management of alcohol‐associated liver disease [26]. In a U.S. cohort of 213 LT recipients, whole‐blood PEth ≥ 20 ng/mL identified recent moderate‐to‐heavy use with 73% sensitivity and 96% specificity versus a composite reference of self‐report and collateral confirmation, while 29% of patients with positive PEth denied drinking [27]. Similarly, in a Spanish cohort of 136 LT recipients, 46% of patients labelled as abstinent by standard clinical evaluation had positive PEth results [28]. Compared with PEth‐negative patients, those with positive results were younger, had a shorter post‐LT duration, more frequent liver test abnormalities, and higher rates of documented nonadherence to follow‐up.

These findings underscore PEth's utility in transplant medicine. PEth objectively documents abstinence and detects relapse with high sensitivity and specificity, providing transplant teams actionable information to guide counselling, tailor surveillance, and intervene earlier when drinking recurs. When incorporated into pre‐ and post‐LT monitoring alongside routine clinical assessment, PEth may help preserve graft function, improve patient survival, and support equitable allocation of scarce donor organs.

## 5 Future Directions for Research

**FIGURE 1:**
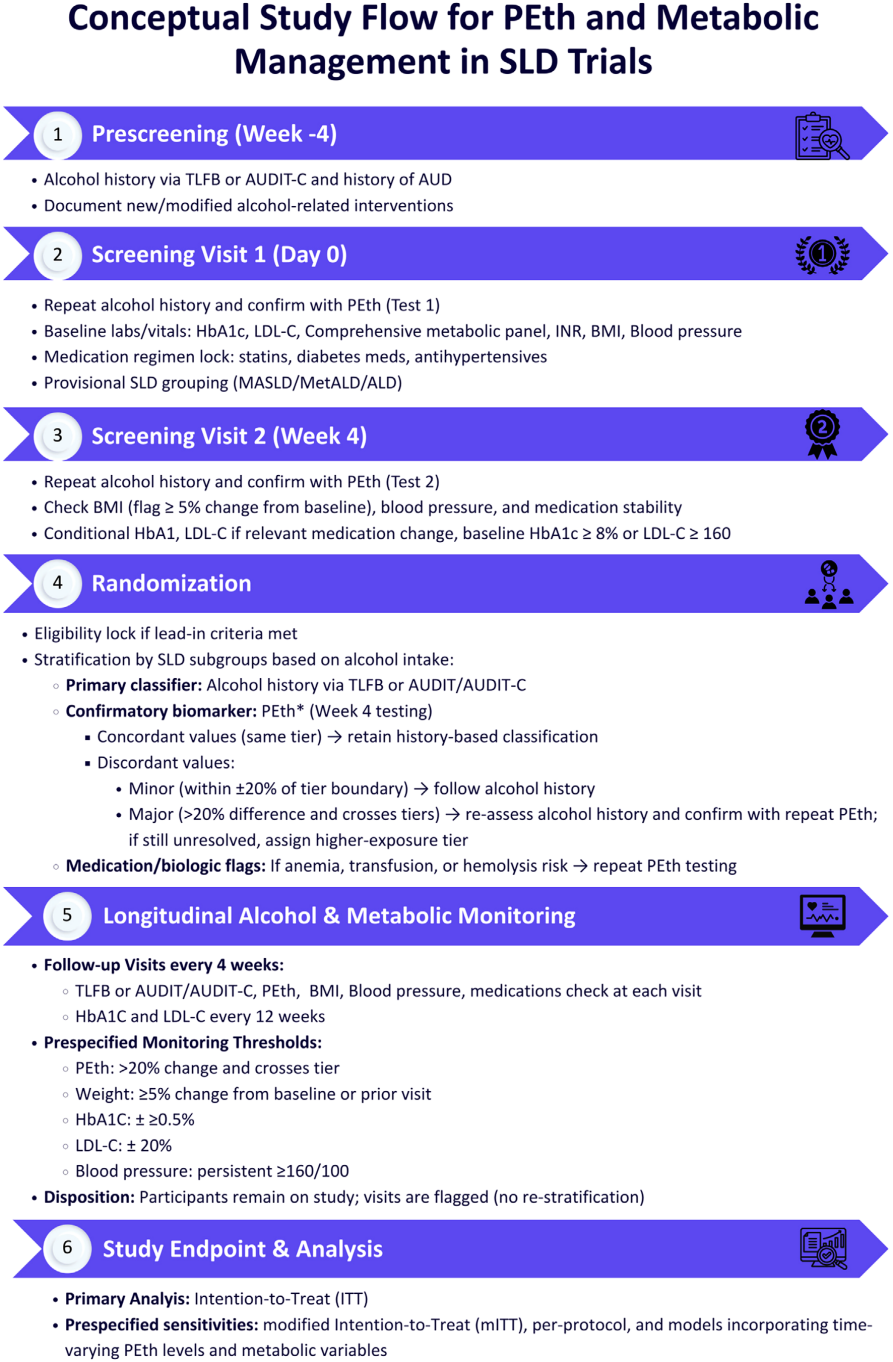
Conceptual study flow for phosphatidylethanol and metabolic management in steatotic liver disease trials. *PEth thresholds are protocol‐specific and must be pre‐specified and justified in each trial's protocol/analysis plan. ALD, alcohol‐associated liver disease; AUD, alcohol use disorder; BMI, body mass index; HbA1c, haemoglobin A1c; INR, international normalised ratio; ITT, intention‐to‐treat; LDL‐C, low‐density lipoprotein cholesterol; MASLD, metabolic dysfunction–associated steatotic liver disease; MetALD, metabolic dysfunction and alcohol‐associated liver disease; mITT, modified intention‐to‐treat; PEth, phosphatidylethanol; SLD, steatotic liver disease; TLFB, Timeline Followback.

Although PEth is the most sensitive and specific biochemical marker of recent alcohol consumption, each result reflects only a snapshot in time. In clinical trials, a single baseline measurement risks misclassifying patients, given the variability of alcohol intake in SLD. To strengthen trial integrity, we propose a structured 4‐week lead‐in screening period that balances the trade‐offs of additional cost, screening failures, and attrition against the benefit of selecting a true cohort with stable alcohol exposure at baseline (Figure 1). This design directly addresses a key limitation in the alcohol clinical research field—the wide gulf between intention‐to‐treat (ITT) and per‐protocol populations due to loss to follow‐up. By requiring both a prescreening history and two sequential PEth assessments, the approach ensures accurate risk stratification and SLD subgroup assignment (MASLD, MetALD, ALD), while also enriching for participants who are more likely to remain adherent. Those who complete the lead‐in become the ITT cohort, which avoids penalising the study for early attrition and enhances downstream retention and interpretability. Specifically, the process includes:

- Prescreening: Obtain a detailed patient history of alcohol use over the prior 12 weeks (3 months) using structured self‐report instruments.
- Screening Visit 1: Perform baseline whole‐blood PEth testing.
- Screening Visit 2 (4 weeks later): Repeat PEth measurement to confirm consistent exposure.

Once enrolled, serial PEth monitoring (e.g., monthly) remains essential. Alcohol intake fluctuations can obscure true drug effects on histologic endpoints (biopsy) and noninvasive measures such as liver stiffness (kPa) or MELD score, while complicating attribution of adverse events (AEs) and serious adverse events (SAEs) to the study drug versus alcohol. Incorporating upper and lower PEth thresholds as eligibility maintenance criteria may help standardise exposure during follow‐up.

Other disease modifiers—including weight, glycemic control, dyslipidemia, and hypertension—must also be systematically measured and stabilised, as they independently affect SLD outcomes. From a pragmatic and ethical standpoint, participants should be asked to maintain baseline lifestyle habits with respect to alcohol intake, diet, and exercise throughout the study. While a minimal brief alcohol intervention at enrollment may be reasonable, intensive therapy to reduce intake should be avoided, since the trial's goal is to evaluate investigational drugs against a consistent background of alcohol exposure. Placebo arms should receive identical minimal interventions to avoid bias. Importantly, this design also reflects regulatory realities: the FDA approves drugs based on anticipated real‐world use, and it is essential to know whether agents are truly safe and effective in MetALD patients who continue to drink.

An additional consideration is the mechanistic profile of investigational drugs. Some emerging therapies—such as GLP‐1 receptor agonists or agents targeting FGF‐21—can simultaneously reduce alcohol intake and exert direct hepatoprotective effects (anti‐inflammatory, antifibrotic) [29, 30]. This dual mechanism raises unique trial design challenges. Granular longitudinal biomarker assessments (alcohol biomarkers such as PEth, alongside metabolic measures including HbA1c, lipids, and blood pressure) are needed to disentangle whether improvements arise from reduced drinking, direct hepatic effects, or both. Moreover, outcome design must align with the drug's mechanism of action:

- For dual‐mechanism agents, composite endpoints that capture both alcohol‐use outcomes (e.g., reduction in heavy drinking days, sustained low‐risk use) and liver‐specific outcomes (histology, noninvasive fibrosis markers) may be most appropriate.
- For liver‐specific agents, endpoints should emphasise hepatic histologic and functional outcomes, with alcohol exposure stabilised in the background.

Taken together, a prescreening history plus a 4‐week paired PEth lead‐in, followed by repeated whole‐blood PEth testing and standardised monitoring of metabolic cofactors, offers a practical and rigorous trial design. By further tailoring outcome selection to the mechanistic class of the investigational agent—and aligning with FDA's expectation of real‐world applicability—this approach recognises alcohol as a dynamic co‐factor rather than a rigid classifier, reduces misclassification, and enhances both endpoint interpretability and participant retention.

## 6 Conclusion

Alcohol consumption is a central modifier of disease biology and outcomes across the SLD spectrum, underscoring the need for accurate, objective assessment. Whole‐blood PEth has emerged as the most reliable short‐term biomarker of recent alcohol use, with the potential to complement physician‐assisted self‐report and validated questionnaires both in clinical and research settings. By assessing exposure over ~2–4 weeks and enabling serial measurements, PEth quantifies alcohol as a continuous co‐factor—informing clinical care, refining SLD subclassification, and strengthening surveillance after liver transplantation. In clinical trials, pre‐randomization and on‐study PEth can document and help maintain stable background exposure, improve risk stratification, aid AE/SAE attribution, and enhance endpoint interpretability without relying solely on rigid categorical labels. Embedding PEth within standardised care and trial protocols offers a practical path to better patient outcomes and more credible therapeutic evaluation in SLD.

## Funding

Dr. Cotter is supported by the American Association for the Study of Liver Diseases (AASLD) Clinical, Translational and Outcomes Research Award (CTORA) and National Institute on Alcohol Abuse and Alcoholism (NIAAA) K23AA031310 grant. The views expressed do not necessarily represent the views of the NIAAA.

## Conflicts of Interest

The authors declare no conflicts of interest.

## Data Availability

The authors have nothing to report.
